# Opportunities for Live Cell FT-Infrared Imaging: Macromolecule Identification with 2D and 3D Localization

**DOI:** 10.3390/ijms141122753

**Published:** 2013-11-19

**Authors:** Eric C. Mattson, Ebrahim Aboualizadeh, Marie E. Barabas, Cheryl L. Stucky, Carol J. Hirschmugl

**Affiliations:** 1 Department of Physics, University of Wisconsin-Milwaukee, 1900 E Kenwood Blvd, Milwaukee, WI 53211, USA; E-Mails: emattson@uwm.edu (E.C.M.); abouali2@uwm.edu (E.A.); 2 Department of Cell Biology, Neurobiology and Anatomy, Medical College of Wisconsin, 8701 Watertown Plank Road, BSB building 428, Milwaukee, WI 53226, USA; E-Mails: mbarabas@mcw.edu (M.E.B.); cstucky@mcw.edu (C.L.S.)

**Keywords:** infrared spectromicroscopy, flow cell, raster scanning, microtomography, transflection, scattering, deconvolution, *Thalassiosira weissflogii*, sensory neurons

## Abstract

Infrared (IR) spectromicroscopy, or chemical imaging, is an evolving technique that is poised to make significant contributions in the fields of biology and medicine. Recent developments in sources, detectors, measurement techniques and speciman holders have now made diffraction-limited Fourier transform infrared (FTIR) imaging of cellular chemistry in living cells a reality. The availability of bright, broadband IR sources and large area, pixelated detectors facilitate live cell imaging, which requires rapid measurements using non-destructive probes. In this work, we review advances in the field of FTIR spectromicroscopy that have contributed to live-cell two and three-dimensional IR imaging, and discuss several key examples that highlight the utility of this technique for studying the structure and chemistry of living cells.

## 1. Introduction

Infrared (IR) spectroscopy is an incisive, non-destructive and non-invasive tool for examining live cells, and allows one to identify the chemical species present within the sample under investigation. IR light detects distinct chemistry based on absorption “fingerprints”, providing inherent contrast, without disturbing the sample even under adverse conditions, conferring access to vital, *in vivo* information. IR spectroscopy is a mature field, yet, more recent schemes coupling it with microscopy, implemented as raster scanning and widefield microspectroscopy and spectro-microtomography measurement schemes provide chemically and spatially resolved 2D projections or 3D images of samples. These methods are poised to make significant contributions to the newer directions embraced by scientists in live cell imaging. Many of these state-of-the-art experiments have been facilitated by IR radiation extracted from storage rings or synchrotrons [1,2] (radiation is emitted when swift charged particles are accelerated by a magnetic field). For example, IR absorption measurements performed on live single cells can be used to probe the distribution of chemistry within the system in 2D projection [3–6] and 3D images [7], and monitor the changes in chemical concentration under different stages in their metabolic cycle [8] throughout development, after injury or disease, or in response to varying environmental stimuli [9]. This may reveal novel information about cell surface topography or complexes or individual molecules important in cellular signaling or transduction of external stimuli.

The use of Fourier transform infrared (FTIR) spectromicroscopy for the study of living cells was recently reviewed by Quaroni [10] and with an emphasis on the use of the synchrotron source by Holman [11]. Both of these works provide valuable information on practical matters such as measurement optics and approaches to aqueous measurements. The present review emphasizes several new developments within the past few years, including different approaches to microfluidics, focal plane array (FPA)-based, broad-spectral bandwidth imaging 3D tomographic imaging. Issues regarding measurement schemes, sample preparation, data analysis, and spatial resolution limits are discussed. A number of recent examples that highlight aspects of the field, the type of information obtained, and the potential of the technique are presented.

Electromagnetic radiation, including IR light, drives the motion of electric charges in matter. If the natural time scale of any oscillations of the charges in a molecule is close to the period of the electromagnetic radiation shining on the system, a condition known as resonance occurs. Like an adult timing his pushes on a child’s swing to coincide with the motion of the swing, a driving force having the same frequency as the system’s natural frequency efficiently couples to and excites the oscillation. Near resonance, therefore, IR light is efficiently absorbed by the system, allowing the identification of the frequencies of low-energy (1–500 meV) excitations found in the sample. These excitations may involve nuclear motion, such as vibrating molecules, ions, or radicals.

As a practical matter, IR spectroscopy has found its widest application in identifying the chemical compounds present in an unknown sample by the virtue of frequencies of IR light the sample absorbs. Since the resonance condition occurs over a narrow range of frequencies, which differs for different compounds (*i.e.*, lipid *versus* carbohydrate functional groups), the exact frequency of the absorbed light provides a characteristic signature of the molecules, ions, or radicals present in the sample. Extensive gas-phase and solution phase studies have identified these “fingerprints” for a host of chemical compounds, which can be used in interpreting surface and interface data. For example, the vibrational stretching motion of a triple-bonded CO unit (such as found in CO gas) absorbs IR light at 5.70 × 10^13^ Hz. Similarly, CO weakly bound to a single atom on a solid surface absorbs IR light at 5.53 × 10^13^ Hz. The analytical capabilities of IR spectroscopy are invaluable for identifying chemical composition within complex, often heterogeneous biological systems.

Einstein won a Nobel Prize in Physics (1921) for showing that the energy carried by electromagnetic radiation is directly related to the frequency of its oscillation [12]. Thus, IR spectroscopy allows the determination of the energy of the excitations it probes, and thereby sheds light on the microscopic origin of the excitation. For example, identifying what functional groups exist within cells—based on their known vibrational excitations—can provide insight into how fixation and arsenic induce changes in biomolecules, or to assess native cellular heterogeneity at the chemical level, as detailed below. The basics of IR absorption spectroscopy are described briefly below, and additional details can be found in [13]. Recent advances in instrumentation, including the design of spectrometers and detectors and the development of new sources, however, provide the means to enhance significantly the capabilities of this mature field, and are described below. As a result of these developments, more complex systems, such as single cells, can be evaluated at high resolution. Here we highlight the recent application of the use of broadband, bright IR synchrotron radiation (SRIR) raster scanning (RS) and widefield (WF) spectromicroscopy and spectro microtomography in living cells in order to showcase/illustrate multiple, diverse opportunities using IR imaging in live, native cells to understand cellular properties that may mediate function.

### 1.1. IR Absorption Spectroscopy

For IR spectroscopy, the process of interest is absorption. IR photons are absorbed by vibrations that induce dynamic dipoles, oscillations in the density of electrons or electron charge due to atomic motion (the electrons follow the motion of the nuclei). The natural oscillating frequencies of molecules are related to the masses of the displaced atoms and the strength of their respective chemical bonds. A molecule consisting of N atoms has a number (3N-5 for linear molecule or 3N-6 for all others) of distinct vibrational modes that are also referred to as normal modes, which each have a distinct absorption frequency. Thus, a “fingerprint” or series of absorption bands with specific vibrations associated with the functional groups for a specific macromolecule are identified, and an absorption spectrum is a plot that shows how well different frequencies of light couple to excitations for the macromolecule.

**Figure 1:**
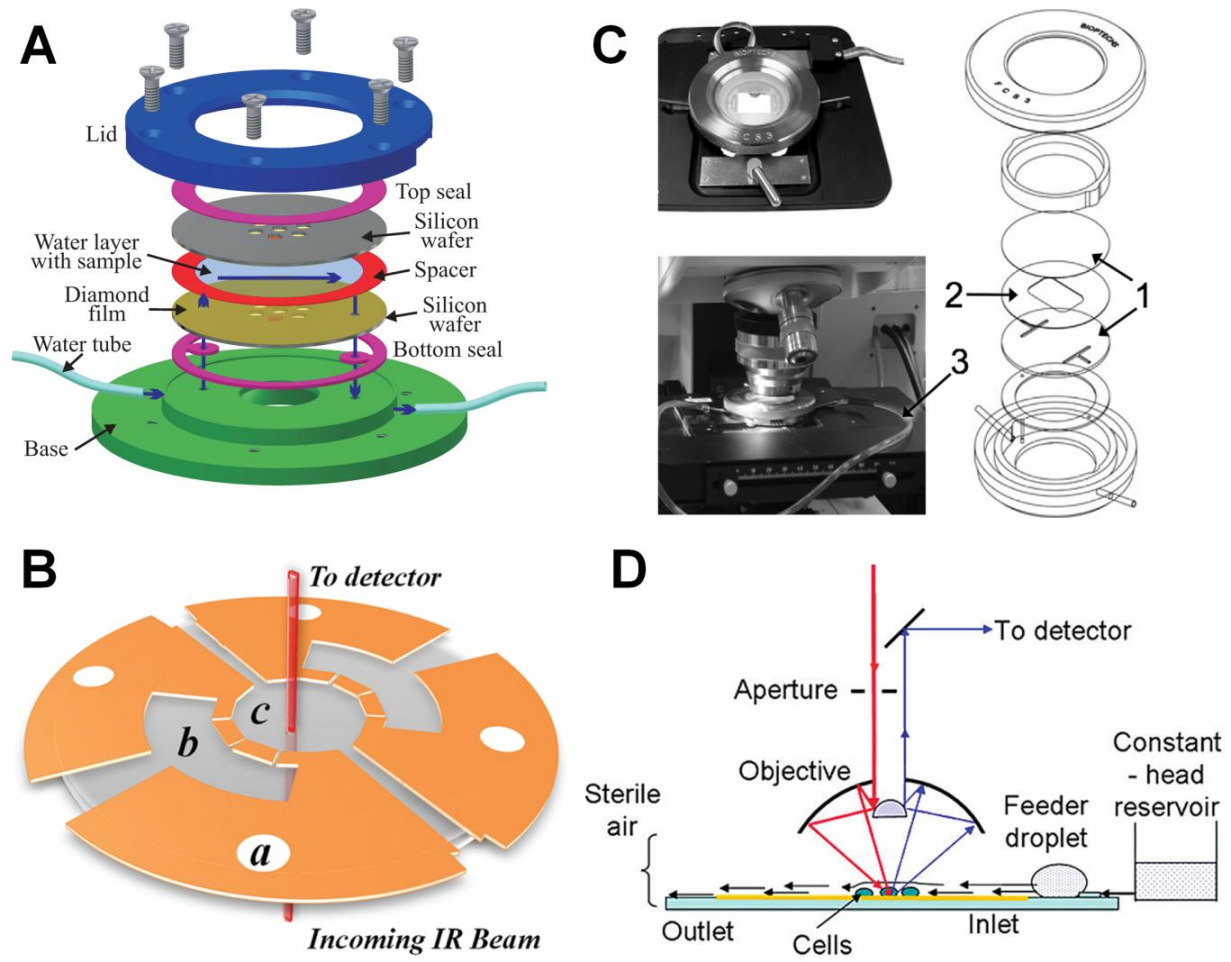
Different schemes of chambers for sustaining living cells. (**A**) Demountable liquid flow cell using submicrometer thick diamond windows [3]; (**B**) Fully-sealed microfluidic chamber employing 1 and 2 mm thick CaF_2_ windows as the top and bottom windows, separated by an 8.5 μm photoresist layer (Reprinted with permission from [6]. Copyright 2012 American Chemical Society); (**C**) Demountable liquid flow cell employing 2 mm thick CaF_2_ windows as the substrate and lid for the cell (Reprinted with permission from [14]. Copyright 2010 Elsevier); and (**D**) Open channel microfluidic design employed by Holman *et al.*, consisting of 10–15 μm deep microchannels embedded onto a Si chip with controlled inlet and outlet pressures, (Reprinted with permission from [5]. Copyright 2009 American Chemical Society).

An absorption spectrum is commonly plotted as (see Figure 1): Absorbance *A vs.* frequency ν, which is related to the transmittance by *A =* −log *T* [It is conventional to convert the units for frequency ν from Hz (s^−1^) to wavenumbers (cm^−1^) by dividing ν by the speed of light *c*]. Absorbance curves exhibit peaks at energies where the sample has absorbed energy from the incident beam. Functional groups within molecules absorb IR light when they are in resonance with the incident radiation and lead to peaks in the absorption spectrum. The frequencies are dependent on the masses of the atomic constituents and the bonding strength and can therefore be used to identify the functional groups. The absorption strength can also be correlated with the concentration of the functional groups and ideally increases linearly for a wide range of concentrations. Obtaining concentrations from 2D projection measurements relies upon having a controlled sample thickness or a precise knowledge of the sample thickness at the point being measured since absorption depends on both concentration and path length. Furthermore, for such parameters to be extracted, the sample must be of uniform thickness over the dimension sampled by a given measurement (e.g., 10 μm for a 10 × 10 μm^2^ aperture size). Obtaining relative concentrations for a given sample or sampling area is reliable if the dynamic dipole strengths of a particular mode is known. Concentration measurements for irregular samples are becoming more feasible with the development of spectromicrotomography experiments due to the controlled thickness of evaluation with the voxel cube.

### 1.2. IR Spectrometers, Sources, and Detectors

Over the years, there has been continual improvement in spectrometers, window materials, and data processing. A comprehensive review of these items can be found in Siesler and Salzer [15]. The most notable development for spectrometers is the development of commercially available, reliable, Fourier transform IR (FTIR) instruments. They are based on measuring interference patterns between two beams from the same source, and employing fast mathematical algorithms to extract full mid-IR spectral information (and therefore broad chemical information) from one rapid measurement.

Recently, there have been major developments in new sources of IR radiation. Traditional IR lab-based sources are hot filaments (Carbon-Tungsten globars) providing IR radiation similar to the lamps that keep food hot in serving lines. This light spans a wide range of frequencies [500 to 4000 cm^−1^, where cm^−1^ is a frequency unit (wavenumber) and refers to the number of waves in one centimeter], and therefore can provide information about a wide range of chemistry in samples. However, the globar source characteristics make it challenging to use it for more demanding experiments. Newer IR sources include radiation emitted from a storage ring (SRIR) and lasers. These sources are high-brightness sources, which means they emit radiation that originates from a point-like source, and the light is therefore confined to a well-defined, small beam (e.g., like laser beams). This property is required to measure spatially resolved data with spatial resolution comparable to the wavelength of the light (<1 to 10’s μm). Laser sources are typically monochromatic, providing light at one frequency or a narrow range of frequencies (narrow-band, such as Quantum Cascade Lasers [16,17], that can be tuned over narrow bandwidths), while the present paper will focus on results from the storage ring sources that are inherently broadband, providing access to a wide range of chemical signatures simultaneously.

Parallel to the source development timeline, IR detectors have also undergone transformations. Traditional detectors for IR spectroscopy employed single element detectors based on Mercury Cadmium Telluride (MCT) IR sensors, and provide one absorption spectrum for a sample at a given time. Recent advances include the development of large IR detector arrays [Focal Plane Arrays (FPA)] for parallel detection of 1000’s of pixels of data, simultaneously.

### 1.3. Synchrotron Radiation FTIR (SR-FTIR) Raster Scanning and Widefield Spectromicroscopy

FTIR spectromicroscopy combines the chemical specificity of mid-IR spectroscopy with diffraction-limited spatial resolution, and is becoming an increasingly utilized modality for non-invasive label-free molecular imaging. An IR absorption spectrum is collected at every pixel within the field of view (FOV), yielding three-dimensional “hyperspectral cubes” or maps of the position-resolved absorbance at each mid-IR wavelength. Two dimensions of the cube represent position on the sample and the third dimension represents the spectral domain and consists of an absorption spectrum. IR chemical images are generated based on the absorption at a particular wavelength/frequency for each pixel in the dataset, allowing for visualization of IR absorption, or equivalently, macromolecular functional groups, over the FOV.

IR spectromicroscopy has recently evolved [18] by several technological advances; first, coupling IR microscopes with SRIR [19] rather than the traditional thermal IR source yields higher signal-to-noise ratio (SNR) than that of the thermal source when using smaller apertures (in confocal experiments) and therefore higher spatial resolution. The tradeoff of this approach is that diffraction-limited imaging at the shortest wavelengths requires a small aperture that reduces the signal for the entire bandwidth, pushing the signal for the longest wavelengths below the detection limit. If a larger aperture is used, spatial resolution for the shorter wavelengths is degraded. Further, the confocal setup has the drawback that image acquisition time ranges from several hours to days, precluding time-resolved measurements such as biochemical kinetics of living cells. The second advance was to replace the single-element detector with a FPA detector (illuminated by a globar) to accelerate data acquisition [20]. Efforts to illuminate FPA detectors with SRIR beams was pioneered by Moss *et al.* [21] and Carr *et al.* [22,23]. They succeeded in illuminating a small portion of the FPA to obtain higher SNR in the illuminated area; however, these small illumination areas meant that only a small subset of the FPA corresponding to a relatively small sample area could be used.

Infrared Environmental Imaging (IRENI), located at the Synchrotron Radiation Center in Madison, WI, USA [24] is the first IR WF-spectromicroscopy system that has been specifically designed and optimized to overcome the SNR-acquisition time-spatial resolution tradeoff by coupling multiple synchrotron beams with a large-FOV FPA detector. This capability enabled spectrochemical imaging for challenging problems such biological and medical applications that require many samplings to collect statistically relevant data and/or high throughput, and analysis of heterogeneous materials at the micrometer scale. In particular, IR imaging of cells *in vivo* is a rapid, nondestructive tool to identify the chemical composition of biological samples for a wide range of physiological and biochemical applications, now realizable with IRENI [2,4,25–38]. Because water is a strong IR absorber [39], benchtop chemical imaging with blackbody thermal sources is limited in attainable spatial resolution and/or SNR when imaging live cells that are immersed in water or require aqueous environments. In this way, use of the bright synchrotron source provides distinct advantages, as it overcomes the SNR problem for thin layers of water, [3,9,40] and recent developments in flow cells [3,5,14,41,42] make these experiments feasible. With the IRENI capabilities reaching biologically relevant spatial and temporal scales, new information on short length and time scales can be obtained in a simultaneous and rapid fashion in living cells or tissue. These developments represent a critical step forward for the field, as now diffraction-limited chemical images can be collected for living cells *in vivo* in a matter of minutes, a critical aspect for the field of living cells which can evolve and move over short time-scales.

## 2. Experimental and Analytical Methods

### 2.1. 2D Projection Raster Scanning and Widefield Spectromicroscopy

#### 2.1.1. Transmission

Most samples are mounted on an IR transparent substrate or maintained in a microfluidic chamber with IR transparent windows. For these situations, measurements are collected by first collecting a background signal that we call *I*_o_, which measures the initial intensity and distribution of the IR source in absence of the absorbing sample. A sample measurement is collected when the sample is introduced. The two measurements are divided, revealing the amount of light transmitted at each wavelength, or alternatively the amount of light absorbed at each wavelength. The latter result is described in the section about IR absorption spectroscopy.

#### 2.1.2. Transflection

In some cases, samples are mounted on reflective substrates. In this case, a background measurement is taken from the bare reflective surface, and the sample measurement is taken from the sample mounted on the same substrate. Care must be taken when interpreting data from these assemblies, as the electric field is not uniform in intensity as a function of distance perpendicular to the surface, and this directly impacts the intensity profiles that are detected [43,44]. In these measurements, the detection of chemical compounds is reliable, and of the electric field, intensity can be accurately predicted. However, for samples with a heterogeneous mixture of chemicals with a thickness of greater than 0.5 μm the relative concentrations of the molecules cannot be accurately determined, since the variation in the electric field and the chemical heterogeneity both contribute to the strength of the absorption bands.

### 2.2. 3D IR Spectral Microtomography

Life science is particularly important to study in three dimensions since essential cellular functions within living tissues such as architecture, mechanical, thermal and biochemical triggers and cell-cell communications are missed by cell culture monolayer models [45]. Imaging methods are required with quantitative biochemical imaging within 3D tissues and 3D cell cultures. Spectro-micro-tomography technique combines FTIR WF spectromicroscopy with computed tomography approaches to greatly enhance the capabilities of both FTIR spectroscopy and tomography by creating a “full color” micro-tomography where colors can be assigned by specific spectral identification or spectral changes. Computed tomography is a well-established approach [46] used to reconstruct cross-sectional slices through an object from the transmitted projection images taken as a function of angle around a single axis of rotation. These slices can be stacked to produce a 3D image of the object, which can then be visualized by a number of methods, including volume rendering or digitally slicing through the sample along any arbitrary plane. Each voxel contains a wealth of information for advanced spectral segregation techniques such as clustering, neural networks, and principle components analysis.

### 2.3. Microfluidics

Two-dimensional (projection) FTIR spectromicroscopy of living cells represents a challenging endeavor due to the need for many cells to be suspended in aqueous media. Water is strongly absorbing in the mid-IR region, and optical path lengths of water greater than 25 μm result in total absorption over a considerable portion of the mid-IR region. Several groups have developed devices to circumvent this measurement difficulty while preserving the integrity of living cells. These approaches include demountable flow chambers [3,14] and open [5] and closed [6,14] micro fluidic devices. Schematic diagrams of chambers used by several different groups to sustain living cells while performing IR measurements are depicted in Figure 1. In demountable liquid flow chambers, two biocompatible IR transparent windows form the basis of the chamber and are separated from one another by a thin spacer, often teflon, which serves a dual purpose of providing a fixed distance between the top and bottom window as well as sealing the chamber. Nasse *et al.* [3] designed a custom demountable flow chamber (Figure 1A) employing sub-micrometer diamond films or 0.5 mm thick diamond windows supported by Si wafers as the top and bottom windows of the cell. The advantage of the use of thin windows is twofold: first, diamond effectively reduces the effect of chromatic aberrations since its index of refraction is unchanging from the visible to the mid-IR range, and the thicknesses are chosen to minimize the multiple internal reflections from within the windows that produces spectral interference fringes. The design by of the dynamic flow cell by Tobin *et al.* [14] (Figure 1C) has a similar demountable design to that by Nasse but uses 2 mm thick CaF_2_ windows rather than sub-micrometer diamond. CaF_2_ is ideal down to approximately 1100 cm^−1^, where the refractive index starts to change due to the phonon absorption band in the material.

Other groups have used microfluidic fabrication methods to construct flow chambers with precisely controlled spaces between the chamber. Vaccari *et al.* [6] used a 2 mm CaF_2_ window as the bottom layer of the chamber and used ultraviolet photolithography to template a thin (8.5 μm) photoresist layer onto the surface (Figure 1B). This design allowed for multiple chambers to be connected by a porous septum, one chamber containing fresh media and one the target sample. A 1 mm thick CaF_2_ window was employed as the lid of the chamber, and was thermomechanically sealed with a hot press to produce a fully sealed micro fluidic chamber. Tobin *et al.* [14] also designed a chamber for static imaging of cells based on a microfluidic design, but this chamber had the drawback of having a maximum operation time of approximately 1 h due to leakage of the medium outside of the chamber.

Holman *et al.* [5] used microfluidic manufacturing methods to fabricate an open-channel flow chamber for live-cell imaging in transflection mode. This design, depicted in Figure 1D, employs a Si wafer for the substrate etched with square or rectangular micropatterns of 10–15 μm depth. A constant laminar flow was maintained by constant hydrostatic pressure at the inlet and capillary pulls at the outlet. A thin gold film was deposited onto the micropatterns to allow significant reflectivity for transflection IR measurements. While this measurement geometry has the advantage of eliminating any appreciable multiple-reflection interference effects from the windows or between the windows, effects from standing waves fixed at the gold surface can nonuniformly effect band intensities at different wavelengths, adding significant complication to measurement interpretation.

### 2.4. Scattering Effects

Cells or subcellular structures that are similar in size to the wavelength of probing light (2–10 μm) will inherently scatter the incident light, as opposed to just absorbing, reflecting or transmitting the light. This phenomenon is called Mie Scattering and is similar to the diffuse scatter of light from a rough surface or wavy lake on a windy day, when you cannot see a strong reflection of the sun incident upon it. For the measurements discussed in this paper, these scattering effects can impact the spectra, affecting the line shapes and peak positions of absorption bands [47–52], and therefore care must be taken when interpreting results. Sometimes this effect is small, when cells are immersed in a medium that reduces the strength of the scattering. When the scattering impacts the data, there are excellent mathematical predictions and algorithms available to remove these effects from measured data. One particular algorithm that is available to extract absorption information from a measured spectrum is entitled RMieS [47–54] for Resonant Mie Scatter correction. Generally, the algorithm starts with a scatter free reference spectrum (*Z*_ref_) (Matrigel, that is a commercial extra cellular matrix that contains all components expected for tissues/cells), and uses this to extract a scatter free spectrum from the raw spectrum (*Z*_raw_). It is assumed that *Z*_raw_ is the superposition of *Z*_ref_ and a number of scattering curves that are determined from the broadband signal in each spectrum from the hyperspectral cube. The resonant correction seeks to remove the impact of the real and imaginary part of the index of refraction of the scatterers. The mathematical background of RMie scattering correction is described in detail elsewhere. Other algorithms are based on Maxwell’s Equations, and predicting the propagation of light through the microscope and sample. They are excellent at predicting the outcomes for a known sample morphology and constituents [50,51], but the inverse problem, to extract the morphological and chemical information from the sample measurement is at present not available with this exact formulation.

### 2.5. Large Data Sets

Hyperspectral cubes of data from IR, RS and WF spectromicroscopy data sets provide thousands to hundreds of thousands of spectra, and can be analyzed with statistical approaches, in addition to traditional image analysis. Traditional image analysis of IR hyperspectral data includes generating grey or false-color scale images based on integration or peak intensity for a given absorption peak, or ratios between two integration or peak intensities. The former results are representative of both the thickness of samples and concentrations of the functional groups that exist in the sample, while ratios remove effects due to thickness. Statistical analysis is a critical tool, since the variations in spectra can be very subtle (It is critical to consider the sample preparation carefully, so as to not remove chemistry from the system and therefore reduce the statistical difference between diverse regions of the system. For living cells, this should be less of a challenge than for tissues that are frequently fixed with chemical treatments that remove chemical components from the biological materials [2]. However, if cells are cultured, then potential effects of injury or culture media need to be considered).

Statistical approaches include principal component analysis and clustering methods. Typically, the spectral data is pre-processed to apply statistical analysis, including calculating spectral derivatives (first and second order), spectral smoothing, and normalization [55–58]. Once these steps have been taken, even subtle differences in spectra can be identified through standard statistics. For principal component analysis, the first component (PC1) is representative of the average intensity at each spectral data point across the spectral range. The second component (PC2) is representative of the variation from the first component. Thus, the whole spectral range is evaluated at once and provides the clues to the differences in the macromolecular constituents that correspond to a given sample. For example, for two sets of data from a cell exposed to different stresses that have been reduced to principal components, the largest variations across the spectral range in PC1 and PC2 can help identify which absorption bands, and therefore what chemical changes are contributing to the different response of the system. Alternatively, established clustering approaches that seek to find similarity among data, in this case seek similarity among the spectra in a data set across the full spectral range. In this case, the similar spectra can be averaged together providing measures of the average spectral responses from the classes of spectra that can then be compared. For large variations in data, the spectra for different classes will be very distinct. Further analysis may be necessary to highlight the differences for small variations. The clustering approaches are also helpful with the image analysis, since the pixels associated with the clustered spectra can easily be identified. Thus, one can achieve “cluster-based images”. A combination of these different approaches is used in the examples provided below.

### 2.6. Diffraction-Limited Spatial Resolution

When the effective sampling area (pixel or aperture size) is sufficiently small, the spatial resolution is limited only by the wavelength of the probe light; *i.e.*, it is diffraction-limited. Stelzer [59] showed that the spatial oversampling required to achieve diffraction-limited spatial resolution was such that 8 sampling points must be acquired over the dimension of the point-spread-function (PSF) that describes the response of the imaging system by a point light source. When this criterion is met and the spatial resolution is diffraction-limited, the resolution that can be obtained is ultimately determined by the numerical aperture (NA) of the objective used, since this determines the characteristics of the PSF. The relationships between spatial oversampling, NA and spatial resolution were recently explored by Mattson *et al.* [30,60] within the context of imaging biological cells and tissues. This work indicated that both NA and spatial oversampling are critical in obtaining optimal spatial resolution, which for the WF imaging setup can match that of the confocal setup in an approximately 1 min acquisition time. Furthermore, it was recently predicted [25,61] and demonstrated [30,36,60] that deconvolution of the instrumental PSF from measured data can further enhance the spatial resolution beyond the diffraction limit. The interested reader is referred to these works for more detail. A review of the highest reported spatial resolutions reported throughout the mid-IR for the WF and RS geometries is given in [30]. The relationships between spatial resolution and acquisition times are further discussed in [4].

## 3. Examples

### 3.1. 2D Projection RS and WF FTIR Spectromicroscopy

#### 3.1.1. Transmission

##### 3.1.1.1. Biochemical Alterations Induced by Different Fixation Protocols on U937 Leukemic Monocytes (RS Spectromicroscopy)

2D projection IR spectromicroscopy of living cells in transmission mode is an emerging technique that has shown considerable promise to investigate chemistry and structure of living cells. A major advantage of this technique is that it circumvents the need for methods such as labeling with fluorescent tags and fixation. In pioneering work, Vaccari *et al.* [6] addressed one of the most fundamental questions surrounding the motivation behind live cell imaging, namely, the difference in chemistry between fixed and living cells. In this work, spectra of living monocytes (U937 cell line) were compared to monocytes prepared for IR spectromicroscopy using different fixation protocols, including formalin fixation, ethanol fixation and air-drying. Spectra of living U937 cells were acquired using the fully-sealed microfluidic device described previously [6]. Spectra of the fixed cells were collected from densely packed cellular monolayers plated onto bare 2 mm thick CaF_2_ windows.

**Figure 2:**
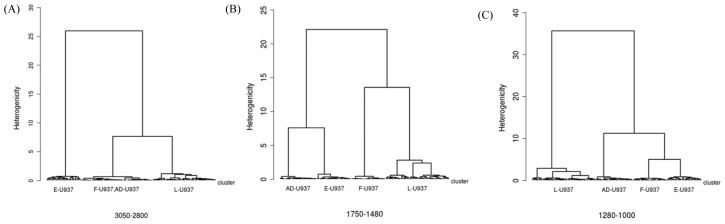

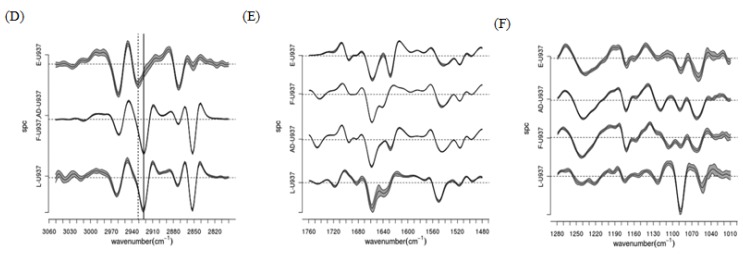
(**A**–**C**) Dendrograms of the classification of vector normalized second derivatives of spectra from L-, F-, E- and AD-U937 monocytes as obtained by HCA (Euclidean distances, Wards’ algorithm) in the regions of (**A**) lipids; (**B**) proteins-phospholipids; and (**C**) nucleic acids-carbohydrates; (**D**–**F**) centroids of the major classes identified by HCA in the (**D**) lipids; (**E**) proteins-phospholipids; and (**F**) nucleic acids-carbohydrates regions. Line thickness is proportional to standard deviation, (Reprinted with permission from [6]. Copyright 2012 American Chemical Society).

To qualitatively address differences in chemistry of the four differently treated cell types, band integrals arising from different functional groups were calculated and hierarchical clustering based on second-derivative spectra was performed. Figure 2 shows dendrograms and centroids of the clustering performed in the regions from 2800 to 3050 cm^−1^ (A,D), 1470 to 1760 cm^−1^ (B,E), and 1010 to 1280 cm^−1^ (C,F) for living (L-U937), ethanol fixed (E-U937), formalin-fixed (F-U937) and air-dried (AD-U937) cells. Consider first the C–H stretching region (2800–3050 cm^−1^). In this spectral region, the centroids for the L-U937, F-U937 and AD-U937 appear in many respects similar, while those of the E-U937 cells demonstrated frequency shifts consistent with increased cellular membrane permeability and disorder. In addition, the lipid and phospholipid content of the E-U937 cells was significantly decreased due to the action of ethanol as a solvent for the lipids, extracting them from the cellular membrane. In the spectral region due to absorption from proteins (Amide I and II absorption bands), the L-U937 and F-U937 show robust similarity, which indicated that formalin fixation largely preserves the cellular protein secondary structure and integrity. In contrast, the E-U937 and AD-U937 show two extra components in the second derivative spectra that were attributed to protein misfolding and consequent aggregation and precipitation. Despite the similarity between the F-U937 and L-U937 spectra, the overall amide content in the F-U937 was depleted, indicating that the formalin fixation results in an overall loss of protein as compared to the physiological state. The most dramatic changes were observed in the fingerprint region, where the vibrations due to the components of nucleic acids and carbohydrates are found. The PO_2_ asymmetric stretching mode is sensitive to nucleic acid conformation, with components at 1220 and 1240 cm^−1^ due to B-helices and A-helical forms of DNA and RNA [62,63]. In the L-U937 spectra, both features were observed; the 1240 cm^−1^ band was assigned by the authors to the A form of RNA and the 1220 cm^−1^ band to the B-helical form of both DNA and RNA. In the E-U937 and F-U937 spectra, the PO_2_ asymmetric stretching mode is observed at 1242 and 1237 cm^−1^, respectively, and appeared less resolved from the band at 1220 cm^−1^ in both cases. Based on these differences in the PO_2_^−^_asym_ region, it was proposed that air-drying, ethanol and formalin fixation all contribute to at least partial denaturing of the nucleic acids.

Thus while formalin-fixation and air-drying are effective in preserving overall protein and lipid conformation, nucleic acids and carbohydrates are strongly modified in both of these protocols. Ethanol fixation even more severely perturbs cellular chemistry, modifying not only the carbohydrates and nucleic acids but also overall lipid content, protein content, and protein secondary structure.

##### 3.1.1.2. Arsenic-Induced Changes to Intracellular Biomolecules in Live Leukemia Cells (RS Spectromicroscopy)

IR Spectromicroscopy is gaining momentum in the fields such as cancer diagnosis and fundamental disease research and therapy. While many studies have compared IR spectra of normal and diseased cancerous tissues [64–66]*in situ* IR spectromicroscopy of living cells has recently shown potential to make a valuable contribution in cancer therapy research. Munro and colleagues investigated the effect of As_2_O_3_, the primary ingredient in the leukemia drug Trisenox™, on leukemia cells [67]. While Trisenox was initially found to be successful in treating 60%–85% of acute promyelocytic leukemia (ACL) patients who had previously relapsed after conventional treatment, the exact mechanism behind its success is not precisely known, and could be relevant in the treatment of other types of cancer. Therefore, Munro *et al.* [67] performed time-dependent microspectroscopy experiments on living ACL cells using the demountable flow cell developed by Tobin, described previously [14].

**Figure 3:**
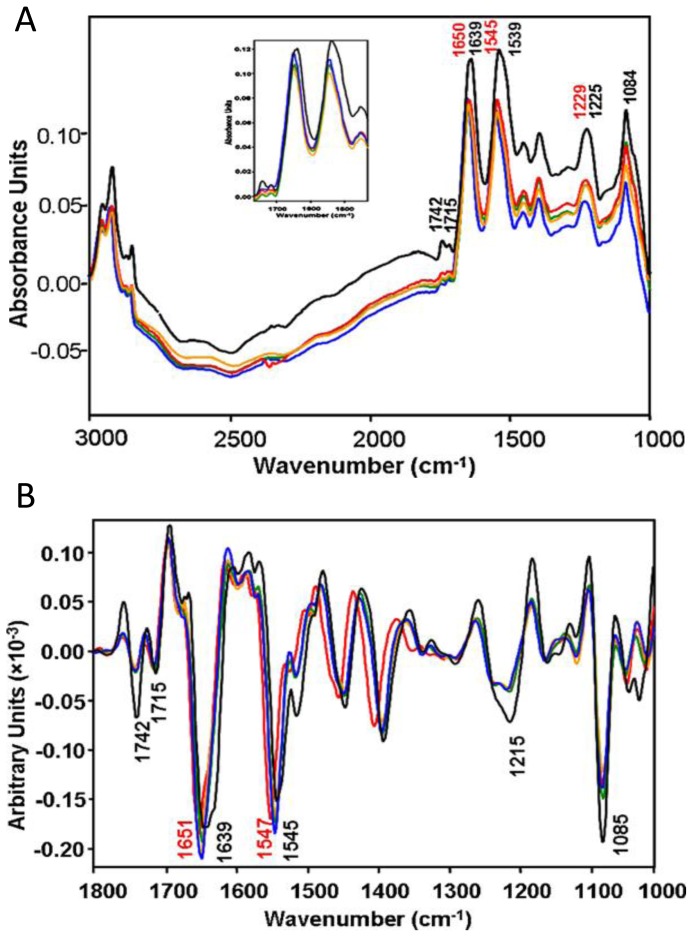
(**A**) IR spectra of living leukemia cells taken *in situ* without the introduction of sodium arsenite (**black**) and after introduction of sodium arsenite for 40 min (**orange**), 60 min (**blue**), 100 min (**green**) and 120 min (**red**). The inset shows a focus on the carbonyl ester and amide region; and (**B**) shows second-derivatives from (**A**). The derivative-spectra clearly show shifts in the amide I (1600–1700 cm^−1^) and amide II (1500–1600 cm^−1^) bands between the control and arsenite-treated cells. (Reprinted with permission from [67]. Copyright 2010 Elsevier).

IR measurements were performed at the Australian Synchrotron on living ACL cells suspended in media with or without sodium arsenite (100 mM concentration). ACL cells measured in media without sodium arsenite were used as a control, and the time dependence of cellular chemistry of the ACL cells treated with sodium arsenite was observed. Spectra of approximately 20 cells were collected and averaged for every time point. The resulting spectra are shown in Figure 3A, with Amide I second-derivative spectra shown in Figure 3B. Here the black spectra are the controls measured with no exposure to arsenite, and the orange, blue, green, and red are from cells exposed to arsenite for 40, 60, 100 and 120 min, respectively. The spectra shown in Figure 3A demonstrate an undulating baseline due to Mie scattering effects, although the spectral features are minimally affected due the fact that the most severe intensity modulations lie in the transparent IR region (1800–2700 cm^−1^) for these cells and therefore resonant Mie scattering and dispersion artifacts do not affect the amide I band. From these spectra, several immediate observations were drawn: first, the bands due to PO_2_^−^ symmetric and asymmetric stretching are smaller in the arsenite-treated cells relative to the Amide I bands and as compared to the control cells. The time-resolved measurements indicated that after 40 min arsenite exposure, the PO_2_^−^ content decreased dramatically, but then increased after 100–120 min exposure. In addition, a significant decrease in the intensity of the carbonyl ester band, associated with phospholipids, was observed. In the amide region, significant shifts of the amide I and II bands were observed, as is evident in the second-derivative spectra; in the untreated control cells, the amide I peak position at 1639 cm^−1^ was assigned to a majority of β-sheet protein conformations. The arsenite-treated cells, for which the amide I peak position shifts to 1650 cm^−1^, suggest changes in protein conformation from β-sheet to either α-helix and/or random coil structures. These changes were observable after 40 min exposure to arsenite, indicating a rapid effect on the secondary protein structure.

From these changes, a number of conclusions were drawn regarding the effect of arsenite on the chemistry of the leukemia cells. Importantly, the band at 1715 cm^−1^ that is present in the control cells is dramatically diminished in intensity following exposure to arsenite. This band is commonly associated with DNA in leukemia cells and is a marker of base-pairing. The observed reduction in intensity indicated that arsenite effectively diminished the overall quantity of detectable DNA and was ascribed to DNA cleavage during apoptosis. This behavior, in conjunction with the observed time-dependence of the phosphate absorption, was proposed to be indicative of the progression of apoptosis. Second, the decrease in the lipid carbonyl bands was attributed to a loss of membrane integrity, which the authors further confirmed using Trypsin Blue staining. Lastly, the most significant changes noted by the authors was the shift in the amide I band indicating a transformation of the protein secondary structures. Such a dramatic change indicates a direct interaction between the arsenite and protein; previous X-ray absorption spectroscopy studies showed that intracellular arsenic was tris-sulfur coordinated and was likely bound to protein, which could be linked to the changes in secondary structure [68]. This study shows the high potential of IR microspectroscopy in monitoring chemical changes in cells *in situ* and, in particular, understanding the dynamics of drug-cellular interactions at the molecular level.

##### 3.1.1.3. Subcellular Imaging of Chemical Moieties in Sensory Neurons (WF FTIR Spectromicroscopy)

Chronic pain is a considerable burden, affecting nearly a third of adults in the United States [69], and chronic pain conditions, arise from altered signaling in both the peripheral and central nervous system [70,71]. The organization of how the peripheral nervous system senses painful stimuli and the alterations that occur during chronic pain states has been a central topic in the research of acute and chronic peripheral pain [72,73]. However, one of the major complexities for determining the underlying mechanisms of pain is that dorsal root ganglia (DRG) neurons, which comprise sensory nerves, are exceptionally heterogeneous, in that there are many functionally distinct subpopulations, some of which respond to light touch, heat, cold or endogenous or exogenous chemical stimuli. One major way used to subclassify DRG neurons is by size as a loose approximation for the degree of myelination. Nerves that are highly myelinated can conduct signals rapidly and often relay information about light touch or gentle warming/cooling [71]. Myelinated Aβ and Aδ nerve fibers typically have large-diameter (LD, ≥27 μm) cell bodies, whereas small-diameter (SD, <27 μm) sensory neurons generally correspond to the unmyelinated, C-fibers *in vivo* [71,74]. To investigate these different subpopulations, IR chemical imaging of living sensory neurons was performed at the IRENI beamline at the Synchrotron Radiation Center (UW-Madison). Cultured DRG sensory neurons were isolated and cultured as described previously [74,75].

**Figure 4:**
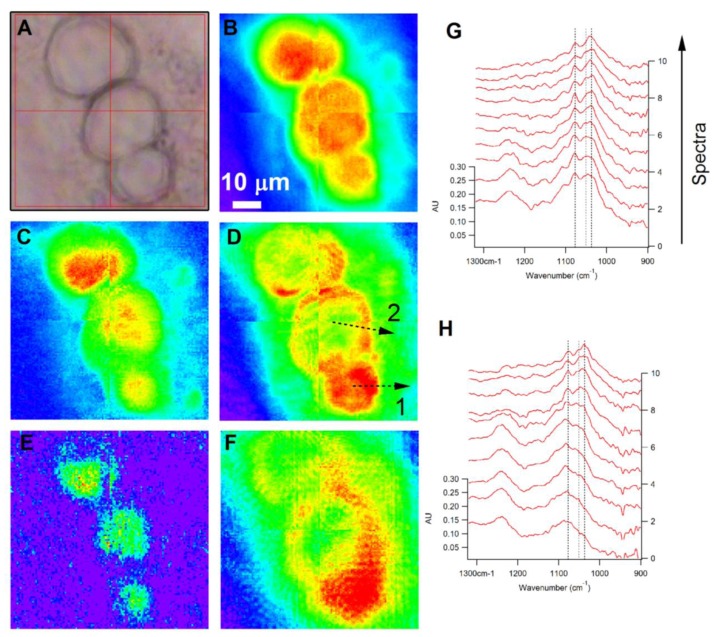
Visible (**A**) and Chemical (**B**–**F**) images of different sized DRG neurons. Chemical images were generated by integrating with a baseline in the regions (**B**) 1605–1705 cm^−1^; (**C**) 2800–3000 cm^−1^; (**D**) 3000–3600 cm^−1^; (**E**) 1718–1765 cm^−1^; (**F**) 993–1134 cm^−1^; (**G**) and (**H**) show sequences of spectra taking from equally spaced points along the lines marked 1 (**G**) and 2 (**H**). The sequence proceeds along the profile, with the spectrum from the beginning of the profile at the bottom of the stack and the end of the profile at the top. The scale bar of Figure 4**A**–**F** is 10 μm.

Figure 4 shows visible (A) and chemical (B–F) images of an aggregate of 3 DRG neurons of varying diameters (74× magnification). The diameters of the cell bodies (as determined from the visible image) are, from top to bottom: 26, 27 and 21 μm. The chemical images are generated by computing integrated area under the absorption bands between (B) 1705–1605 cm^−1^ C=O stretching of amide functional groups); (C) 3000–2800 cm^−1^ (C–H stretching), (D) 3600–3000 cm^−1^ (O–H and N–H stretching from carbohydrates, protein and water), (E) 1765–1718 cm^−1^ (C=O stretching of phospholipid ester) and (F) 1134–993 cm^−1^ (C–O stretching and C–O–H deformation of carbohydrates). The chemical image of the cells shown in Figure 4B, generated from the amide I absorption band, indicates a homogeneous distribution of amide, with the greatest intensity focused within the center of the cells and concentrically decreasing toward the periphery of the cells. A similar trend is observed for the integrated images generated from the absorption bands characteristic of lipid (Figure 4C) and phospholipid (Figure 4E). In contrast, there is a different trend for the localization of carbohydrates in the small and large diameter neurons (Figure 4F). In the two larger cells (26 and 27 μm diameter), the carbohydrate signatures show the highest integrated intensity in an annular pattern that outlines the regions of the greatest amide and hydrocarbon absorption. A similar pattern is observed in Figure 4D, indicating that the predominant signal in this spectral region arises from the O–H stretching vibrations of carbohydrates. The smallest diameter cell (21 μm), on the other hand, demonstrates carbohydrate spatial distributions that closely mirror those of protein and lipid. Another interesting feature, shared by all 3 of the cells in the field of view, is the substantial carbohydrate absorption that extends well outside the main cell body, which appears to be unique for sensory neurons. This region outside the cell body has strong carbohydrate features, and weak CH and amide absorption bands, indicating a much higher relative concentration of carbohydrates in this region. This observation cannot be an imaging artifact attributed to the longer wavelength of the C–O and C–O–H vibrations used to image the carbohydrates. The integrated image of the O–H stretching region shows the same trend thus confirming that this observation is real. Sensory neurons are known to express numerous glycoproteins, such as [74–76] those that contain α-d-galactose and bind isolectin B. This carbohydrate-rich extension of the cell can most likely be attributed to the glycoprotein residues present on the surface of the cell membrane that extend outside of the cell.

To illustrate the cell chemistry in more detail, in Figure 4G,H we show sequences of spectra extracted along profiles from the largest and smallest of the neurons (27 and 21 μm, respectively). The locations used to extract the spectral sequences are indicated by the dashed lines in Figure 4D; the stack in Figure 4G,H comes from profile (1,2) in Figure 4D; from bottom to top, the spectral stacks come from equally spaced locations beginning in the center of the cell and extending to the exterior region at the end of the profile. First, consider the spectral series extracted from the SD neuron (Figure 4G, profile 1). This spectral region (900–1300 cm^−1^) contains primarily the features due to phosphate and carbohydrate functional groups. The asymmetric and symmetric stretching features of the PO_2_^−^ ion are observed at 1244 and 1087 cm^−1^, respectively. The C–O stretching and C–O–H deformation modes of carbohydrates produce a series of bands in the 1000–1200 cm^−1^ region, including strong bands at 1040, 1053, 1082, 1110 and 1150 cm^−1^. The profiles indicate that the phosphate groups are concentrated predominantly within the interior region of the cell, while the carbohydrates are delocalized and extend outside of the main cell body. At the outermost point from which spectra were extracted, there appears to be almost no concentration of phosphate, yet the carbohydrate signatures still appear strong, and permit analysis of the carbohydrate frequencies unobscured by the presence of the PO_2_^−^ symmetric stretch. In the region from 1000 to 1200 cm^−1^, this series of peaks and their relative intensities are similar to the profile observed for glycogen; however, the positions of the strongest bands are somewhat shifted compared to that of glycogen. For example, the strongest band in the spectrum of glycogen is observed between 1020 and 1030 cm^−1^; however, the spectra in Figure 4G show a maximum value at 1040 cm^−1^, with an additional peak contributing at 1052 cm^−1^, which is not present in glycogen. These differences most likely arise due to the presence of other carbohydrate moieties present within the cell, such as α-d-galactose. When compared to the spectra of pure d-galactose, the carbohydrate spectra in Figure 4H show strong similarity and have the same overall profile, with some minor peak shifts.

In the case of spectra extracted from the profile across the large diameter neuron, very little carbohydrate signature (1000–1200 cm^−1^ region) was observed in the center of the large cell compared to the periphery of the cell, with the dominant contribution in the 1000–1200 cm^−1^ region from the PO_2_^−^ symmetric stretching mode (1087 cm^−1^). Careful analysis of the frequency of this peak as a function of distance from the cell center shows that it is in fact distinct from the carbohydrate peak observed at 1082 cm^−1^, and that within the cell, the two bands overlap. Spectra extracted further toward the exterior of the cell gradually begin to show an absorption lineshape more similar to that observed for the SD cell carbohydrates, and eventually the outermost region demonstrates an absorption lineshape that is the same as the small neuron. The distinct chemical and morphological differences between large and small neurons may contribute to the unique functional properties of these neuronal subpopulations.

##### 3.1.1.4. Time Dependent Macromolecule Changes in Micrasterias Due to Nitrogen Source (RS FTIR Spectromicroscopy)

IR Chemical imaging of living cells in aqueous environments offers a unique method to investigate systems of ecological relevance. For example, several groups have performed *in situ* IR spectromicroscopy, employing the spatial resolution afforded by synchrotron sources, to investigate the physiology of microalgae under environmental stimuli. Understanding the ability of microalgae to adapt to changing environmental conditions is a problem of critical environmental concern, particularly because microalgae are responsible for approximately 50% of total planetary primary productivity [77]. Heraud and co-workers [9] used *in situ* IR spectromicroscopy, employing synchrotron radiation at the SRS in Daresbury, to study the effect of N and P starvation on the chemical composition of living *Micrasterias hardyi.* In this work, time resolved linear mapping of living cells treated with either replete, N-deficient or P-deficient media was performed using the RS, dual-aperture, confocal geometry. Time-dependent imaging experiments were performed on N- or P-deficient cells following resupply of N or P.

**Figure 5:**
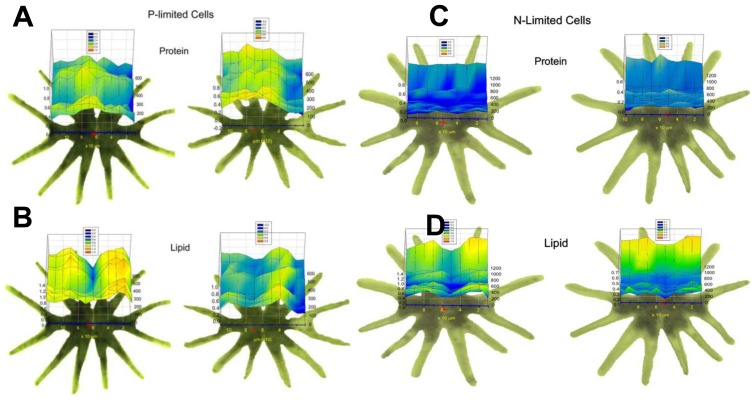
Spatial and temporal changes of lipid and protein concentrations in living *M. hardyi* algal cells subjected to P-starvation (**A**,**B**) and N-starvation (**C**,**D**) following nutrient resupplying. The *x*-axis represents position along the cell and the *y*-axis represents time following resupply of P (**A**,**B**) or N (**C**,**D**). (Reprinted with permission from [9]. Copyright 2006 John Wiley and Sons).

For the measurements, a commercial demountable flow chamber was employed to measure the living cells in media. The results of the time-resolved linear mapping experiments are shown in Figure 5. In each panel, visible images of the cell being measured are shown, with the positions used for the point mapping overlaid. Chemical images generated from characteristic protein and lipid bands are shown for two cells for each condition (*i.e*., during resupply of N or P). Figure 5A shows the protein distribution of two cells subjected to P-deplete media, and Figure 5B shows the lipid distribution of the same cells. Analogous data are shown in Figure 5C,D for cells subjected to N-deplete media. Here the 3-D plots indicate integrated intensity of the line profiles indicate as a function of position on the cell (*x*) and time (*z*). Despite the rather coarse spatial sampling used in these measurements (20 μm × 20 μm aperture size), the different compartments within individual cells were resolved, namely the nuclear region in the center of the cell and chloroplasts at the periphery. For cells in replete media, N-deplete media, and P-deplete media, it was observed that the nuclear region, the region at which the two halves of the cell meet (central region of the linear maps), has elevated protein and decreased lipid relative to the chloroplasts (peripheral regions of the linear maps). The P-starved cells showed a higher lipid content than that of the nutrient-replete cells. Following resupply of P, no change in the overall lipid quantity was observed (Figure 5B). Cells that were starved of N showed a decrease in overall protein content relative to the nutrient-replete cells. Following resupply of N, an increase in the lipid content of the chloroplasts was observed, but no change in the overall protein content was detected (Figure 5C,D). This study demonstrates the potential of IR spectromicroscopy to monitor overall macromolecule content and nutritional status of living cells under controlled conditions.

##### 3.1.1.5. Time Dependent Macromolecule Changes in *Thallasosira Weissflogii* Due to Elevated Carbon Dixide Exposure (WF FTIR Spectromicroscopy)

WF spectromicroscopy was used to study changes in concentrations of carbon containing macromolecules due to environmental stressesby rapidly imaging single *Thalassiosira weissflogii* algal cells maintained in a microfluidic chamber. These data result in a series of temporally-resolved IR images, enabling chemically specific visualization that is unattainable with visible imaging, of on-going chemical processes and morphology in live cells.

Diatoms harvested from batch cultures were exposed to high CO_2_ medium (5000 ppm CO_2_) prior to IR measurements, and then maintained within the microfluidic chamber at fixed temperature (20 °C) in 10 μL of CO_2_ controlled medium fixed with a 15 μm spacer, and continually illuminated with photosynthetically active light.

**Figure 6:**
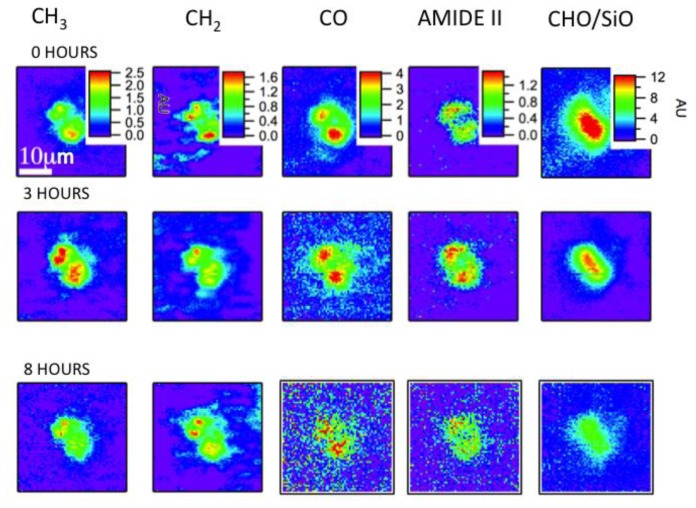
Temporally resolved series of IR images showing distributions of biochemically important functional groups and time dependent changes in the concentrations of several biochemical functional groups for a *Thalassiosira weissflogii* maintained in the flow cell. The images are obtained from data sets collected at 1, 3 and 8 h after exposure to medium containing a high concentration (5000 ppm) of CO_2_. The images are displayed on a rainbow scale, with the red corresponding to the highest detected quantity of the functional group.

The data sets were collected over a span of 8 h. Representative images are shown for the same algal cell in Figure 6, collected at 0, 3 and 8 h time points. The chemical images are generated by integrating the peaks of biomolecular functional groups including saturated CH_3_ and CH_2_ groups relating to C–H stretching (≈3000–2800 cm^−1^), the N–H in-plane bending of amide groups (≈1570–1500 cm^−1^), the C=O functional groups found in fatty acid esters of lipids (≈1760–1710 cm^−1^), the C–O–C and C–C stretching found in carbohydrates (≈1200–1000 cm^−1^). The color scales are preserved for each functional group, thus revealing the variations within the algal cells over time.

**Figure 7:**
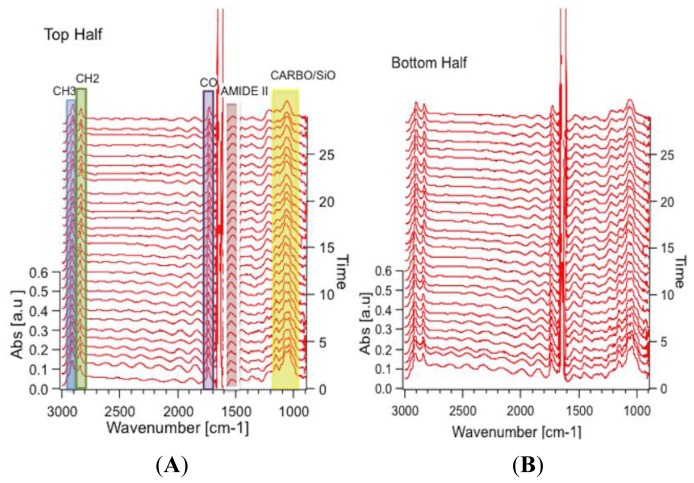
(**A**) Time lapse sequence of spectra created from spatially averaged IR absorption spectra over the top half of the cell in Figure 6. The sequence ranges between *t* = 3 to 8 h. Functional groups are highlighted in the spectral stack; **blue**: CH_3_ (2890–2937 cm^−1^); **green**: CH_2_ (2834–2863 cm^−1^); **purple**: CO (1710–1756 cm^−1^) from a phospholipid or ester; **red**: amide II (1500–1570 cm^−1^); **yellow**: carbohydrate/silica (1016–1186 cm^−1^); and **(B)** Sequence of spectra created from the same dataset as the spectra stack in Figure 7**A**, spatially averaged over the bottom half of the cell.

Figure 7 shows spectra derived from top and bottom halves of algal cell as a function of time. They show the response of each half of the algal cell to the environmental stimuli, which could be distinct since one half of the cell is the “mother” cell, while the other half is the “daughter” cell. The sequence of spectra range from 3 h into the measuring period (bottom of the stack) to the end of the measuring period (top of the stack). Twenty nine spectra covering 300 min of data collection are shown, and are taken approximately 10 min apart. The most obvious changes in the spectral sequence and the IR images are seen in the carbohydrate/silica spectral band. Clearly, the intensity of this band changes from the beginning to the end of the experiment as is evident from the change in the color rendering of the images from the initial time point to the images generated from the time point taken at 8 h. Second, the spectral signature clearly changes its shape, with the peak at 1040 cm^−1^ getting smaller while there are minimal to no changes in the peak at 1080 cm^−1^. The former peak is associated with carbohydrate functional groups, while the latter is associated with silica Si–O functional groups. Further changes are observed for the CH_3_ and CH_2_ functional groups, in both distribution and overall intensity, but the other functional groups did not show such clear changes. No changes are expected for the SiO functional group, but changes are expected in at least some of the carbon containing macromolecules, as observed. This proof-of-principal experiment demonstrates the capability of following such changes *in vivo* with sufficient time resolution to capture the changes in real-time.

#### 3.1.2. Transflection

##### 3.1.2.1. Protein Phosphorylation in Single PC12 Cells during Neuronal Differentiation (RS FTIR Spectromicroscopy)

Holman and coworkers [78] employed *in situ* IR spectromicroscopy to study the process of protein phosphorylation during neuronal differentiation in living PC12 cells. PC12 cells are known to differentiate and exhibit many neuron-like phenotypes [79] following stimulation with nerve growth factor (NGF). Since the differentiation and phosphorylation of this system is well understood [80,81], it represents an ideal model system in which to probe the phosphorylation of living cells with IR spectromicroscopy and identify IR-specific markers of protein phosphorylation in living cells.

The cells were prepared by incubation with NGF-containing medium (or NGF-free culture medium for control samples) for predetermined time periods and plated onto gold-coated microscope slides. The slides were placed in an environmental chamber with 200 nm-thick Si_3_N_4_ windows to facilitate a transflection measurement while maintaining constant relative humidity to sustain the cells. Recall that caution is advised when interpreting intensity changes and when using transflection geometries, due to the varying intensity of the electric field perpendicular to the sample surface. Since the strength of the absorption band is directly proportional to the amount of material present, the electric field intensity, and the strength of the change in the electron distribution in the molecule, the varying electric field should impact the interpretation of the concentration trends. In this work, while the sample preparation and environmental control are key components for this experiment, and the spectra clearly show the presence of important chemistry for samples prepared under varying conditions, care must be taken for the interpretation. As long as the cells are maintained with similar profile, which is significantly smaller thickness than the shortest wavelength of light (3 μm), then the findings will be robust. This is possible for some of the reported measurements, although unlikely to be unchanging across the entire measurement time frame.

**Figure 8:**
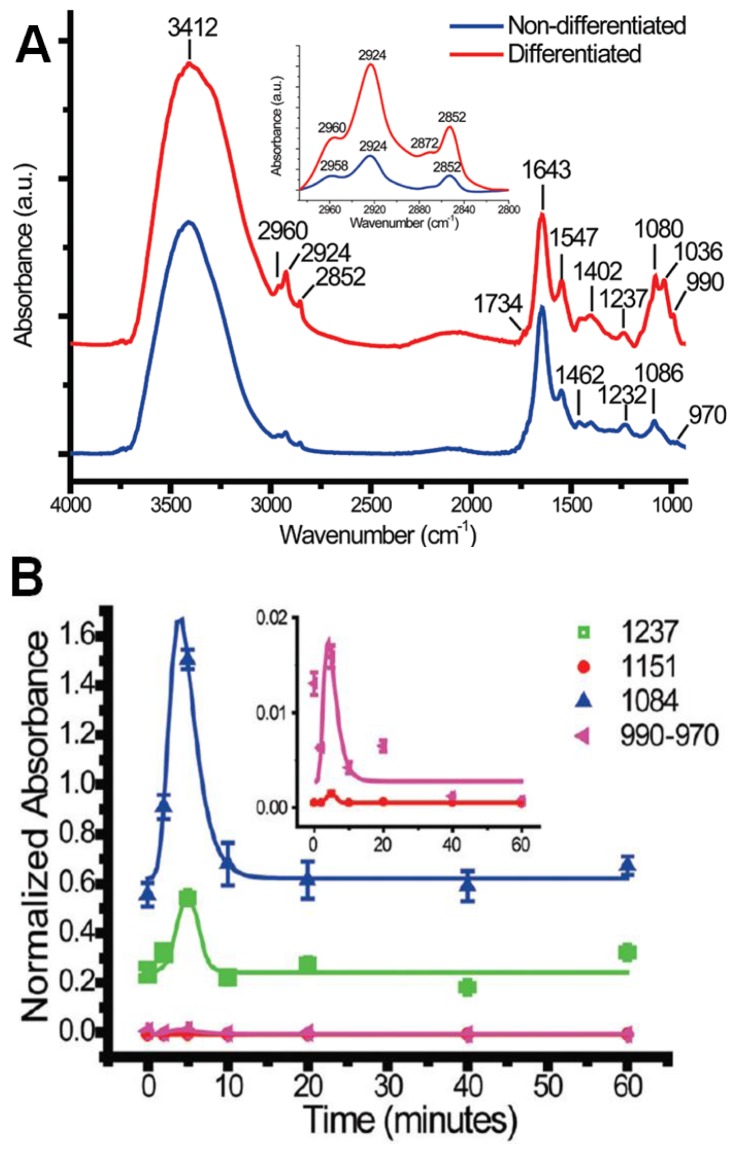

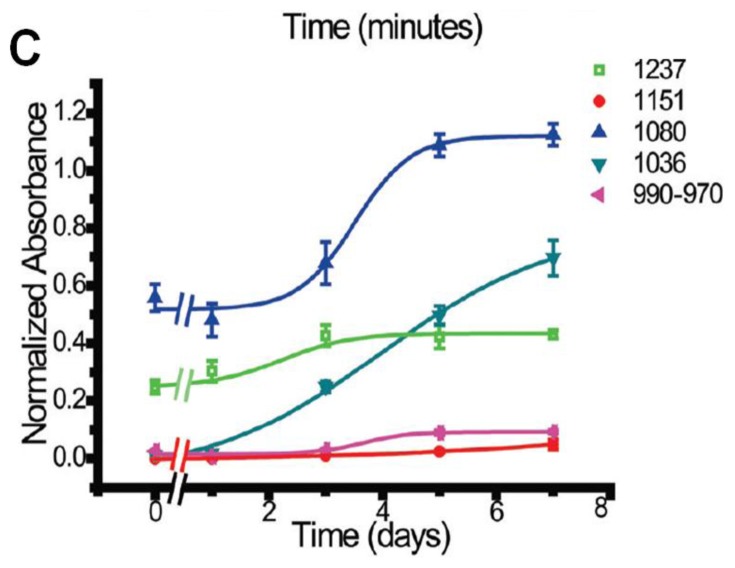
(**A**) Comparison of IR absorption spectra of control (**blue**) and NGF-treated (**red**) PC12 cells; and (**B**,**C**) Time absorbance of bands in the fingerprint region from short-term (**B**) and long-term (**C**) NGF-treatments. (Reprinted with permission from [78]. Copyright 2012 American Chemical Society).

As reported, when IR spectra of cells that were not subjected to any NGF-treatment were compared to those of fully differentiated NGF-treated cells (Figure 8A), a number of differences between the two cell types were observed. First, the NGF-treated cells showed a higher relative absorbance of the peaks associated with lipid (2800–3000 cm^−1^) and phospholipid (1734 cm^−1^), indicating an increase in the lipid-to-protein ratio. The authors also noted that the methylene (CH_2_) bands increased much more than those of the methyl (CH_3_) groups. The most significant changes, however, were observed in the fingerprint region in which the characteristic vibrations of phosphate groups and carbohydrates are found. Phosphorylation was found to cause a dramatic increase in intensity of the bands at ~1237, 1151, 1080, 1036 and 970–990 cm^−1^. In particular, the bands at 1237 and 1080 cm^−1^, ascribed to the PO_2_^−^ asymmetric and symmetric stretching modes, and bands from 970 to 990 cm^−1^ to PO_4_^2−^, showed significant increase and were attributed to phosphorylation of protein and possibly also DNA.

To more closely examine these changes in chemistry during both the early and late stages of differentiation, cells treated with NGF for fixed time periods were examined. To explore early-stage chemistry, cells with 0, 2, 5, 20 and 60 min of NGF-treatment were measured, and cells with 1, 3, 5 and 7-day NGF-treatments were measured to explore late-stage chemistry. Based on the measured spectra, the time-dependent integrated absorption of the bands in the fingerprint region was evaluated (Figure 8B,C). In the early-stage measurements, the bands associated with spectral changes due to phosphorylation were found to increase rapidly at the 5 min time point, and then recover to levels just slightly higher than those of the control at the 10 min time-point onward. This was postulated to indicate a partial reversibility of the chemical changes due to the phosphorylation process during the early-stages of NGF-treatment. In the long-term measurements, the bands at 1151, 1080, 1030, and 970–990 cm^−1^ showed substantial increases over the course of the 7 day measurement period, while the 1237 cm^−1^ band showed only a mild increase. It was noted that the largest increases took place during day 3, at which point scanning electron micrographs indicated the formation of neurites. The positive correlation between, in particular, the 1237 and 1080 cm^−1^ bands and the 970–990 cm^−1^ band was taken as evidence that these bands may be used as spectral markers for protein phosphorylation.

### 3.2. 3D Spectral Microtomography

#### 3.2.1. Macromolecular Architecture of a Colony of Stem Cells

Pluripotent stem cells can assume nearly any functional cell type of an organism, and cell genotype, phenotype and function can change rapidly and unpredictably over time. Most characterization approaches rely on sample manipulation to gain information beyond morphology and are incapable of analyzing intact 3D cell aggregates that are known to be crucial for the maintenance of stem cell state [82]. Recently, 2D vibrational spectroscopy has been applied to study stem cells and several reports have noted a significant spectroscopic change in bands associated pluripotent *versus* multipotent cells [83,84] and between multipotent cells and derived hepatocytes [85]. The utility of vibrational spectroscopy in the future would benefit greatly from the 3D spectral microtomography technique and associated preservation of crucial cell–cell and cell–extracellular matrix interactions.

**Figure 9:**
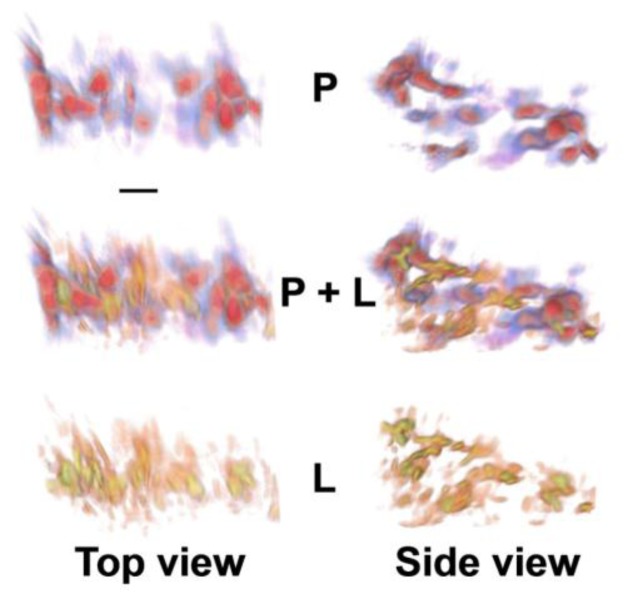
Top and side views of 3D protein and lipid distributions for an embryoid body colony of stem cells. Volume renderings from reconstructions for the protein (P) amide I absorption band [1,650 cm^−1^; blue-red; (**top**)] and lipid (L)-specific bands [2,850 cm^−1^; orange-yellow; (**side**)] reveal three layers of cell bodies with inhomogeneously distributed lipids. The two spectral region reconstructions are shown independently and superimposed (P + L) to provide context for the images. Scale bar, 10 μm. (Reprinted with permission from [7]. Copyright 2012 American Chemical Society).

Here an IR spectro micro tomographic data set of an intact, mouse embryoid body (EB) was obtained. EBs, 3D aggregates of pluripotent stem cells, were harvested and held in a polyamide microloop holder for 3D analysis. At this stage of formation, the EB contains primarily pluripotent stem cells with a smaller fraction of differentiating cells of multiple types. A reconstruction of the Amide 1 spectral region is shown in Figure 9 (top row, blue corresponds to low levels and red to high levels). A reconstruction of the CH peak at 2800 cm^−1^, corresponding to lipids, is shown in Figure 9 (bottom row, yellow corresponds to low levels and orange to high levels). The CH functional group distribution was observed around many of the amide distributions, but was not present uniformly. Based on literature findings noted above, it was hypothesized that the heterogeneous lipid distribution might correspond to differentiating *vs.* pluripotent or progenitor cell types. Future iterations of FTIR spectro microtomography should include the option of fluorescence imaging for known markers of potency to establish links between macromolecular signatures gleaned from FTIR with differentiation state. This technique has great promise not only for stem cell screening but also for better understanding the biochemical structure of differentiating stem cells in their microenvironment.

## 4. Conclusions

There are many new opportunities for IR live cell imaging in multiple dimensions, including spatial in 2D projection and 3D tomographic imaging, and temporally with time-dependent imaging, as presented here. Importantly, synchrotron sources that offer bright IR radiation make it feasible to obtain chemically specific images without damaging or interfering with cell function. The marriage of these sources with pixelated detectors for WF spectromicroscopy has made many of these studies possible. In addition, different approaches to microfluidic devices to maintain the cells in hydrated, controlled condition have been critical to progress of live-cell FTIR spectromicroscopy. Open channel microfluidic devices that are designed for transflection measurements have the advantage of maintaining a well-defined fluid layer with controllable flow, but suffer from undesirable standing wave artifacts that complicate quantitative interpretation and preclude accurate evaluation of relative concentrations. For this reason, closed-channel transmission geometry chambers are more desirable. CaF_2_ windows are excellent substrates for developing microfluidic chambers with designed structures, and are ideal for measurements between 4000 and 1200 cm^−1^. Below this frequency, however, the index of refraction starts to change quickly and will affect the resulting image quality. Chambers that employ diamond as the window material are another excellent choice that allow for access to the full mid-IR spectral range with minimal change in the refractive index. The controllable environment that can be used for the study of living cells lends itself very well to 2D imaging; at present, though, there is no such device to sustain the cells during 3D tomographic measurements. Future efforts should focus on developing viable chambers for sustaining cells during 3D spectro-microtomography measurements.

This combination of advances make it possible to consider asking and answering more challenging questions with chemical imaging from FTIR spectromicroscopy. In future, coupling with cellular function would be a real advance. For example in somatosensory biology, performing IR imaging in conjunction with calcium imaging or patch clamp electrophysiology would lead to new insights with multimodal approaches.
